# Preliminary Study on the Expression of CLLD7 and CHC1L Proteins in Oral Squamous Cell Carcinoma

**DOI:** 10.1055/s-0043-1768468

**Published:** 2023-06-13

**Authors:** Boworn Klongnoi, Bishwa Prakash Bhattarai, Rachai Juengsomjit, Ounruean Meesakul, Sopee Poomsawat, Kajohnkiart Janebodin, Siribang-on Piboonniyom Khovidhunkit

**Affiliations:** 1 Department of Oral and Maxillofacial Surgery, Faculty of Dentistry, Mahidol University, Bangkok, Thailand; 2 Department of Clinical Dentistry, Walailak University International College of Dentistry, Walailak University, Bangkok, Thailand; 3 Department of Oral and Maxillofacial Pathology, Faculty of Dentistry, Mahidol University, Bangkok, Thailand; 4 Department of Anatomy, Faculty of Dentistry, Mahidol University, Bangkok, Thailand; 5 Department of Advanced General Dentistry, Faculty of Dentistry, Mahidol University, Bangkok, Thailand

**Keywords:** CLLD7, CHC1L, immunohistochemistry, oral squamous cell carcinoma, tumor suppressor proteins

## Abstract

**Objective** This study aimed to preliminarily evaluate the expression of two putative tumor suppressor proteins, including chronic lymphocytic leukemia deletion gene 7 (CLLD7) and chromosome condensation 1-like (CHC1L) proteins in oral squamous cell carcinoma (OSCC).

**Materials and Methods** Expression of CLLD7 and CHC1L proteins was analyzed in 19 OSCC and 12 normal oral mucosa (NOM) using immunohistochemistry. The percentage of positive cells and intensity of staining were semiquantitatively assessed and expressed with an immunoreactive score. The number of positive cells at various subcellular localizations was evaluated and presented in percentages. The immunoreactivity scores and percentages of positive cells at various localizations were compared between the normal and OSCC groups with statistical significance at *p* -value less than 0.05.

**Results** According to immunohistochemical analysis, the immunoreactivity scores for both CLLD7 and CHC1L were higher in NOM than those of OSCC. Analysis of CLLD7 localization revealed predominant nuclear staining at basal and parabasal areas in NOM, whereas more cytoplasmic staining was observed in OSCC. For CHC1L, nuclear staining was prominent in NOM. In contrast, significantly increased plasma membrane staining was detected in OSCC.

**Conclusion** The expression of CLLD7 and CHC1L proteins was reduced in OSCC. Alterations in the subcellular localization of these two proteins in OSCC were also demonstrated. These preliminary results suggest that CLLD7 and CHC1L are aberrantly expressed in OSCC. The precise mechanisms of these putative tumor suppressor proteins in OSCC require future studies.

## Introduction

Tumorigenesis involves multiple mechanisms, one of which is the mutations in the tumor suppressor genes. Multiple putative tumor suppressor genes are located in the 13q14 region. 1 The retinoblastoma tumor suppressor gene ( *RB1* ) is also located in this region and is mutated in a variety of tumors. 2 However, in tumors such as epithelial ovarian carcinoma 3 4 and head and neck squamous cell carcinoma, 5 the *RB1* showed normal expression suggesting possible mutations of other potential tumor suppressors that lie in proximity to the *RB1* .

*Chronic lymphocytic leukemia deletion gene 7* ( *CLLD7* ) or *RCC1 and BTB domain-containing protein 1* ( *RCBTB1* ) and *chromosome condensation 1-like* ( *CHC1L* ) or *RCC1 and BTB domain-containing protein 2* ( *RCBTB2* ) are potential tumor suppressor genes located at 13q14.3 telomeric to *RB1* . 6 7 The CLLD7 protein contains RCC1 (Regulator of chromosome condensation 1) and BTB (Broad complex, Tramtrack, and bric-a-brac) domains, whereas the CHC1L protein has a BReast CAncer (BRCA) domain in addition to the RCC1 and BTB domains. 8 RCC1 has essential cell cycle functions and is widely expressed, has nuclear localization signals, and is therefore located in the nucleus during interphase. 9 RCC1 is a guanine nucleotide exchange factor (GEF) for Ras-related nuclear protein (Ran). 10 Since Ran-GEF, that is, RCC1, is in the nucleus, whereas Ran GTPase activating protein (Ran-GAP) is in the cytoplasm, this gives rise to a gradient of the active form of Ran that acts as the driving force behind vectorial nucleocytoplasmic transport. 11 The BTB domain works as an adaptor for cullin-3-based ubiquitin ligases, 12 13 a regulator of gene transcriptions, and conducts gene expression by controlling the chromatin structure. 14

*CLLD7* has been postulated to be a candidate tumor suppressor gene in chronic lymphocytic leukemia and a candidate tumor suppressor gene on chromosome 13q14, which regulates pathways of DNA damage/repair and apoptosis. 6 15 A loss of expression of CLLD7 has been observed in several types of cancers, such as cervical cancer, colon cancer, and leukemia suggesting its role as a potential tumor suppressor protein. 15

*CHC1L* is mapped to the smallest common deleted region on chromosome 13q14. It was found that CHC1L expression was significantly reduced in prostate tumors compared to normal prostate tissues. 16 Low expression levels of CHC1L were also observed in peripheral blood lymphocytes of multiple myeloma patients when compared to those of healthy controls. 17 Recently, all cases of atypical pleomorphic lipomatous tumors showed consistent loss of *RB1* and its flanking gene *RCBTB2* , where multiplex ligation-dependent probe amplification was used to evaluate genetic changes of 13q14. 18 Additionally, reduced expression of *CHC1L* has also been observed in histiocyte-rich neoplasms. 19

This evidence suggests that both CLLD7 and CHC1L proteins play important roles in particular types of tumorigeneses. Nevertheless, none of this has been investigated in oral squamous cell carcinoma (OSCC), which rendered the investigation of the expression of these two proteins in this study.

## Materials and Methods

### Tissue Samples

This laboratory-based observational study was designed and conducted at the Department of Oral and Maxillofacial Pathology. The study obtained approval from the Committee on Human Rights Related to Human Experimentation (MU-DT/PY-IRB 2018/025.1106). The ethical guidelines of the Declaration of Helsinki were followed in this study.

The cases diagnosed as OSCC were selected and retrieved as tissues embedded in paraffin blocks from the Department of Oral and Maxillofacial Pathology. Normal oral mucosa (NOM) tissues were collected from the flaps or leftover tissues after the removal of impacted third molars at the Oral and Maxillofacial Surgery Clinic. Only the samples of NOM that did not show any signs of inflammation on both clinical and histological examinations were included. Hospital charts and histopathology reports were used to retrieve the demographic and clinicopathological data of the participants from whom the NOM and OSCC tissues were collected.

### Immunohistochemistry

Three-µm-thick tissue sections of NOM and OSCC were cut and mounted over aminopropyltriethoxysilane coated slides, deparaffinized, and rehydrated. Sections were incubated with 3% H _2_ O _2_ for 10 minutes to block endogenous peroxidase. Antigen retrieval was performed by heating the sections in citrate buffer pH 6.0 using a microwave oven. After washes with phosphate-buffered saline (PBS) pH 7.6 with 0.1% Tween 20 (PBS-T), the sections were blocked using 5% bovine serum albumin. Primary antibody diluted in commercially available diluent or PBS was applied over the tissue sections. Slides were then kept inside a humidifier and incubated overnight at 4°C. The primary antibody concentration for CLLD7 (ab233533, Abcam, Cambridge, UK) and CHC1L (ab175505, Abcam, Cambridge, UK) was 1:400. On the second day, after draining off the primary antibody, slides were rinsed in PBS-T with gentle agitation. A horseradish peroxidase (HRP)-conjugated secondary antibody (Dako REAL EnVision/HRP, Rabbit/Mouse (ENV), Dako, Denmark) was applied over the sections and incubated for 30 minutes at room temperature in a humidified environment. After thorough washing with PBS-T, the color was developed by incubating with freshly-made diaminobenzidine solution. Sections were then washed for 5 minutes in running tap water and counterstained with hematoxylin before dehydration and mounting. Negative control for each sample was done by replacing the primary antibody with PBS. Sections of mouse brain known to demonstrate nuclear staining were stained at the same run as positive controls for both CLLD7 and CHC1L antibodies.

### Evaluation of CLLD7 and CHC1L Positive Cells

For CLLD7 and CHC1L-stained slides, the epithelial cell that demonstrated golden brown staining in the nucleus, cytoplasm, and plasma membrane was considered positive. Five to eight ﬁelds under a light microscope (400 magniﬁcation) were randomly chosen and photographed. Then, the numbers of positive and total cells in each photograph were counted using the ImageJ program. The percentage of positive cells (positively stained cells divided by the total number of cells counted) was determined. Based on a semi-quantitative assessment suggested by Kaemmerer et al, 20 the percentage of positively-stained cells was categorized into one of the following groups: 0 = no positive cells; 1 = < 10% positive cells; 2= 10–50% positive cells; 3= 51–80% positive cells; and 4= > 80% positive cells. The intensity of staining was also evaluated and classified into four levels: 0: no staining; 1= mild staining; 2= moderate staining; and 3= intense staining. Then, a composite immunoreactivity score (IRS) was obtained for each case by multiplying the category of positive percentage (0, 1, 2, 3, and 4) with the level of intensity (0, 1, 2, and 3). Thus, the IRS ranged from 0 to 12.

Furthermore, the positive staining was categorized based on the subcellular localization of the proteins. At least 500 cells were counted in each case. To minimize the inter-observer variation, two authors examined each slide (SP and BPB) independently. Each photo was reassessed by both SP and BPB whenever the percentage of positive cells showed greater than a 5% deviation.

### Statistical Analysis

The expressions of CLLD7 and CHC1L in NOM and OSCC were compared through the immunoreactivity scores and subcellular localization of the proteins. The Kolmogorov–Smirnov test was used for the test of the normality of the data. Whenever the data showed normal distribution, results were expressed in the mean and standard error of the mean (SEM); otherwise, the median and interquartile range were used. Independent samples *t* -test and Mann–Whitney U test were used to compare the differences in percentages of positive cells and subcellular localization of the proteins. Signiﬁcant diﬀerences were established at *p* -value less than 0.05.

## Results

### Participants' Characteristics

The specimens used for the immunohistochemical analysis of the desired proteins were obtained from 31 participants. In total, 19 OSCC and 12 NOM tissues were included in this study. The general characteristics of the participants from whom the specimens were obtained are summarized in Table 1 .

**Table 1:** General characteristics of the specimens by tissue types

Characteristics	OSCC ( *n* = 19)	NOM (pericoronal tissue of 3rd molar) ( *n* = 12)
**Sex (M/F)**	8/ 11	7/ 5
**Age (mean ± SD); range (years)**	59.84 ± 10.37; 42–80	23.67 ± 5.96; 15–37
**Location (** ***n*** **)**	Lateral tongue (7) Hard palate (4) Gingiva (3) Buccal mucosa (2) Tooth socket (1) Floor of mouth (1) Soft palate (1)	Right mandible (6) Left mandible (3) Right maxilla (2) Left maxilla (1)
**Associated risk factors (** ***n*** **)**	Smoking (2) Ill-fitting denture (2) Alcoholism (1)	None
**Histological grade (** ***n*** **)**	Well-differentiated (9) Moderately differentiated (10)	Not applicable
**Metastasis**	None	Not applicable

Abbreviations: NOM, normal oral mucosa; OSCC, oral squamous cell carcinoma; SD, standard deviation.

### Immunoreactivity Scores for CLLD7 and CHC1L

In our study, all cases of NOM and OSCC showed positive staining for CLLD7 and CHC1L. The average immunoreactivity scores for CLLD7 and CHC1L in NOM and OSCC are shown in Table 2 . The immunoreactivity scores in OSCC for CLLD7 and CHC1L were significantly lower than those in NOM ( Table 2 ).

**Table 2:** Immunoreactivity scores for CLLD7 and CHC1L

Protein	Immunoreactivity score		*p* -Value
	NOM	OSCC	
CLLD7	8.08 ± 0.38 a	5.51 ± 0.68 a	0.003 ^b^
CHC1L	7.8 (7.05, 10.5) ^c^	4.75 (2.4, 8.8) ^c^	0.004 ^d^

Abbreviations: CHC1L, chromosome condensation 1-like; CLLD7, chronic lymphocytic leukemia deletion gene 7; NOM, normal oral mucosa; OSCC, oral squamous cell carcinoma; SEM, standard error of mean.

a mean ± SEM; ^b^ Independent samples *t* -test; ^c^ median (Q1, Q3); ^**d**^ Mann–Whitney U test.

### Pattern of CLLD7 and CHC1L Immunostaining

#### CLLD7

**Fig. 1:**
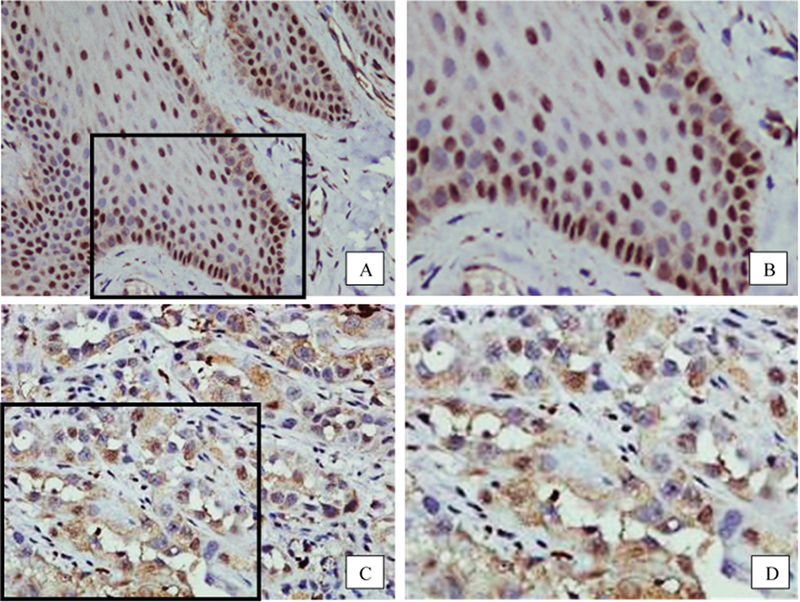
Representative images of immunostaining for chronic lymphocytic leukemia deletion gene 7. **A** (400 X) and **B** (blow-up picture of **A** ): In normal oral mucosa, the majority of nuclear staining is found in the basal and parabasal cell layers. **C** (400 X) and **D** (blow-up picture of **C** ): In oral squamous cell carcinoma, cytoplasmic staining is more commonly observed.

In NOM, all cases showed prominent nuclear staining. The majority of nuclear staining was found in the basal and parabasal layers ( Fig. 1A and 1B ). A small number of cells with cytoplasmic staining and a few cells with membrane staining were also detected in some specimens. Nuclear and cytoplasmic staining were both observed in OSCC. However, nuclear staining was less frequently noted, whereas cytoplasmic staining was more commonly found in OSCC compared with NOM. Membrane staining was not detected in any cases of OSCC ( Fig. 1C and 1D ).

#### CHC1L

**Fig. 2:**
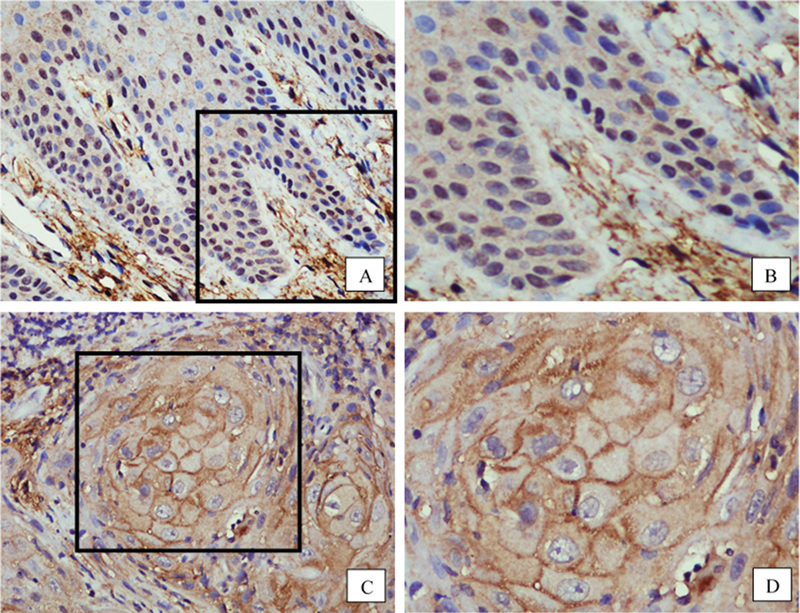
Representative images of immunostaining for chromosome condensation 1-like. **A** (400 X) and **B** (blow-up picture of **A** ): In normal oral mucosa, nuclear staining is predominant. **C** (400 X) and **D** (blow-up picture of **C** ): In oral squamous cell carcinoma, membrane and cytoplasmic decorations are predominant, and nuclear staining is barely observed.

In NOM, numerous cells with nuclear staining were observed in all cell layers of the epithelium. Cytoplasmic as well as membrane staining was also detected, but they were found only in a small number of cells in each case ( Fig. 2A and 2B ). In contrast to NOM, membrane staining was predominant and nuclear staining was barely observed in OSCC ( Fig. 2C and 2D ).

The percentages of subcellular expression of the proteins are shown in Table 3 . Briefly, CLLD7 showed marked nuclear staining in the NOM, whereas cytoplasmic staining was mainly found in OSCC. CHC1L was expressed predominately in the nucleus in NOM, while plasma membrane staining was more prominent in OSCC. Furthermore, we evaluated the differences in the total percentage of CLLD7 and CHC1L positive cells and the subcellular localization of these proteins based on the histological grading of OSCC. We could not find significant differences with respect to the percentage of positive cells and the subcellular localization of the proteins between well-differentiated and moderately differentiated OSCC ( *p* > 0.05).

**Table 3:** Percentage of subcellular expression of CLLD7 and CHC1L in NOM and OSCC

Subcellular localization	CLLD7			CHC1L		
	NOM	OSCC	*p* -Value	NOM	OSCC	*p* -Value
Nucleus	47.25 ± 5.13 a	10.96 ± 1.9 a	< 0.001 ^b^	33.83 (9.03, 50.37) ^c^	0.26 (0.00, 0.39) ^c^	< 0.001 ^d^
Cytoplasm	0.9 (0.15, 5.2) ^c^	10.7 (4.5, 34.6) ^c^	0.001 ^d^	2.57 (0.74, 29.33) ^c^	1.28 (0.00, 17.26) ^c^	0.11 ^d^
Membrane	0.17 (0.00, 1.38) ^c^	0.00	0.02 ^d^	12.00 ± 3.18 a	30.98 ± 6.57 a	0.015 ^b^
Nucleus plus cytoplasm	1.23 (0.01, 4.95) ^c^	2.88 (0.00, 17.68) ^c^	0.389 ^d^	1.96 (0.12, 3.26) ^c^	0.00 (0.00, 0.00) ^c^	< 0.001 ^d^
Nucleus plus membrane	0.13 (0.00, 1.1) ^c^	0.00	0.006 ^d^	3.57 (1.42, 10.66) ^c^	0.00 (0.00, 0.00) ^c^	< 0.001 ^d^

Abbreviations: CHC1L, chromosome condensation 1-like; CLLD7, chronic lymphocytic leukemia deletion gene 7; NOM, normal oral mucosa; OSCC, oral squamous cell carcinoma; SEM, standard error of mean.

a Mean ± SEM; ^b^ Independent samples t-test; ^c^ median (Q1, Q3); ^d^ Mann–Whitney U test.

## Discussion

The characteristics of the patients from whom the tissues for immunohistochemistry were obtained are presented in Table 1 . There is a large difference in the mean age of the OSCC and the NOM groups. This difference reflects the ages at which the diagnosis of oral cancer is made or when a third molar is extracted. According to the hospital-based cancer registry annual report of Thailand, patients above 50 years had the highest incidence of oral cancer, 21 which is consistent with the distribution of the age and characteristics of the OSCC patients in our study. In agreement with our study, Unwerawattana reported that patients aged between 21 and 30 years had the highest frequency of third molar extraction in Thailand. 22

For CLLD7, the overall immunoreactivity score was significantly lower in OSCC compared to the NOM ( Table 2 ). The possible reason for this might be due to the aberration in the *CLLD7* gene causing low expression of the protein. Aberrations of the *CLLD7* gene have been reported in various types of tumors. Using online data mining to evaluate *CLLD7* copy numbers in different tumor cell lines provided by the Wellcome Trust Cancer Genome Project, Zhou and Münger found that out of 776 tumor cell lines, loss of heterozygosity of *CLLD7* gene was reported in 316 cell lines representing 29 different tumors and homozygous deletions were found in two cell lines. 15 Additionally, mRNA expression in cervical and colon cancers, leukemia, and lymphoma was reduced by more than 50% compared to the normal controls. 15 In a microarray-based analysis, Maiuthed et al found that the epigenetic modifications produced by nitric oxide on the *CLLD7* gene resulted in decreased expression of the gene in non-small cell carcinoma of the lungs as compared with the control. 23 Based on these studies, an assumption that the *CLLD7* gene might have been downregulated, causing lower expression of the protein in our OSCC samples, could be made. However, the exact mechanism for the downregulation in OSCC needs to be determined by future studies.

While both nuclear and cytoplasmic CLLD7 staining was observed in NOM and OSCC, OSCC had a much higher percentage of cells with cytoplasmic CLLD7 staining than NOM ( Table 3 ). The observed differences in subcellular localization between OSCC and NOM suggest that the nuclear activity of CLLD7 may be compromised in OSCC. Furthermore, studies have often attributed the subcellular mislocalization of tumor suppressor proteins as one of the mechanisms for tumorigenesis. 24 CLLD7 was abundantly expressed in the cytoplasm of OSCC in our study. Since point mutation might render the protein non-functional or misallocated, we searched for point mutation of *CLLD7* through the TumorPortal database and found that missense mutation and splice-site mutation were documented in head and neck cancers. Thus, there might be a possibility that the mislocalization of CLLD7 has roles for tumorigenesis or that tumorigenesis might have caused the mislocalization. However, future studies are necessary to validate these assumptions.

In this study, one important finding is that the immunoreactivity score for CHC1L in OSCC is significantly lower than that in NOM ( Table 2 ). In addition, in some cases where specimens contained both epithelial dysplasia and squamous cell carcinoma, there was a marked reduction of CHC1L staining in squamous carcinoma cells compared to neighboring cells in the dysplastic area. The reduction in tumor suppressor protein expression during malignant transformation may be caused by allelic imbalance, mutation of the gene causing more degradation of the protein, or epigenetic phenomenon. The *CHC1L* gene is downregulated in various types of tumors. Creytens et al reported a consistent loss of *RB1* and its flanking gene, *RCBTB2 (CHC1L)* , in all cases of atypical spindle cell lipomatous tumors while evaluating genetic changes of 13q14 using multiplex ligation-dependent probe amplification. 18 Similarly, reduced expression of CHC1L was seen in 21 out of 36 primary prostate tumors in a study by Latil et al. The loss of heterozygosity at the 13q14 region, where *CHC1L* is mapped, was seen in 18 of the tumors, and 14 of those tumors showed reduced expression of CHC1L. 16 Furthermore, low expression levels of CHC1L were observed by RT-PCR in peripheral blood lymphocytes of myeloma patients when compared to those of healthy participants. 17 Based on these studies, we speculate that the aberrant *CHC1L* gene may lead to a decreased expression of CHC1L protein in the OSCC samples. However, the exact mechanism of genetic alteration necessitates future studies.

In general, nuclear CHC1L staining was observed in all cell layers of the epithelium in the NOM. Cytoplasmic and membrane stainings were also detected but observed only in very few cells in each case. However, in OSCC, membrane staining was predominant, and there were only very few cells with nuclear staining. Interestingly, strong plasma membrane staining of CHC1L found in this study has not been reported in other tumor types. Protein synthesis usually occurs primarily in the cytosol and is transported to their functional sites. In cancer cells, several mechanisms are responsible for the dysregulation of protein trafficking, leading to the abnormal subcellular localization of proteins. The mechanisms include mutation of protein-targeting signals, dysregulation of transporter machinery, aberrant endocytosis and vesicular trafficking, dysregulation of signal transduction and protein post-translational modification, alteration of protein-protein interactions, and cross-regulation of cancer-related proteins. 24 Until now, the knowledge regarding aberration of CHC1L in OSCC is extremely limited. Exploration through the TumorPortal database revealed missense mutations in head and neck cancers, which may lead to the aberration of the protein. Since CHC1L might have been mislocalized during OSCC transformation, the study on investigation of *CHC1L* gene, CHC1L-interacting proteins, and the cause of the subcellular mislocalization might be encouraged. It is interesting to determine the biochemical basis for the mislocalization and explore the biological consequences of the altered localization of this protein in OSCC.

We acknowledged the limitation of this study in that a low number of NOM and OSCC cases were included. In addition, since this is a retrospective study, only a little information regarding the risk factors of oral cancer was retrieved. Future studies in a larger group of patients with OSCC will be required to elucidate the aberration of these genes as the cause for low expression and changes in the subcellular localization of the proteins. Furthermore, some essential histopathological parameters, such as the depth of invasion and the worst pattern of invasion, could not be evaluated, mainly because a part of the OSCC samples were from incisional biopsy. In future studies, it is essential to retrieve all such histopathological parameters to correlate the protein expression to such parameters. Although the methodology of this study is not new, the expression of CLLD7 and CHC1L and the lower immunoreactivity scores and differences in subcellular localization of the proteins in OSCC compared with NOM have never been reported elsewhere. Therefore, we hope this information could provide insight into the molecular markers for oral cancer. Finally, immunohistochemical analysis of these proteins in oral premalignant lesions would be interesting to identify if the aberration occurs at an early stage of malignant transformation and contributes to oral carcinogenesis.

## Conclusions

The lower immunoreactivity scores and differences in subcellular localization of CLLD7 and CHC1L in OSCC compared to normal oral tissues could lead to a preliminary assumption that the proteins might have been lost during the malignant transformation of the normal tissue. In addition, mutation or aberration of the proteins may render abnormal subcellular localization of the proteins, which leads to improper control of the function of these proteins, thereby accentuating oral tumorigenesis.
